# Prevalence of lameness, claw lesions, and associated risk factors in dairy farms in Selangor, Malaysia

**DOI:** 10.1007/s11250-017-1387-4

**Published:** 2017-08-31

**Authors:** M. B. Sadiq, S. Z. Ramanoon, R. Mansor, S. S. Syed-Hussain, W. M. Shaik Mossadeq

**Affiliations:** 1 Department of Farm and Exotic Animal Medicine and Surgery, Faculty of Veterinary Medicine, Universiti Putra Malaysia, 43400 UPM Serdang, Selangor Malaysia; 2 Centre of Excellence (Ruminant), Faculty of Veterinary Medicine, Universiti Putra Malaysia, 43400 UPM Serdang, Selangor Malaysia; 3 Department of Veterinary Clinical Studies, Faculty of Veterinary Medicine, Universiti Putra Malaysia, 43400 UPM Serdang, Selangor Malaysia; 4 Department of Veterinary Preclinical Sciences, Faculty of Veterinary Medicine, Universiti Putra Malaysia, 43400 UPM Serdang, Selangor Malaysia

**Keywords:** Lameness prevalence, Claw lesions, Dairy cows, Risk factors, Locomotion

## Abstract

The objectives of this cross-sectional study were to estimate the prevalence of lameness, claw lesions, and associated risk factors in dairy farms in Selangor, Malaysia. The sample population was 251 lactating cows from 8 farms assessed for lameness and claw lesions by locomotion scoring and claw assessment respectively while specific animal-based measures were hypothesized as cow-level risk factors. The Wilcoxon rank test and logistic regression were applied to assess the prevalence of lameness, claw lesions, and association with potential risk factors, respectively. The prevalence of lameness was 19.1% ranging from 10.0 to 33.3% while 31.1% of cows had claw lesions and ranged from 16.3–40%. Claw lesions were recorded in 87.5% of the lame cows with highest being those affected with sole lesions (54.2%) and white line disease (61.2%). Overall, the occurrence of overgrown claws, sole lesions, white line disease, and digital dermatitis were 37, 18.2, 10.9, and 8.3%, respectively. More than one claw lesion per cow was present in 71.8% of the affected cows. Lameness was associated with early lactation (OR = 3.3; 95% CI 2–7), injured hocks (OR = 4.8; 95% CI 5-17), and dirty legs hygiene (OR = 2.6; 95% CI 1.3-6.2), whereas presence of claw lesions was associated with dirty legs hygiene (OR = 4.7; 95% CI 4-11) and overgrown claw (OR = 2.7; 95% CI 1.4-5.3). To reduce the prevalence of lameness, farmers need to improve the management of cows with overgrown claw, injured hocks, and cleanliness by establishing routine claw trimming and efficient stall design.

## Introduction

Dairy production is fast growing in South Asia with indications of the highest global demand for milk emanating from the region (FAO 2015). However, the growing intensive management of cattle dairy herds and demand for high milk yield has increased their susceptibility to certain production-limiting conditions such as lameness (Cook et al. 2016). Lameness is a condition characterized by alteration of gait resulting from pain caused by injury to the hoof or limb (Olechnowicz and Jaskowski 2011). Lameness in dairy cows is a welfare problem (Whay and Shearer 2017) and causes economic loss attributed to early culling, treatment, of and reduced milk yield (Green et al. 2014; Thomas et al. 2016). The prevalence of lameness varies amongst herds between regions and countries as cow level prevalence of lameness was 21.98 and 18.9% in western Thailand (Rahman et al. 2014) and Australian dairy herds, respectively (Ranjbar et al. 2016). In India, a recent study reported incidence of lameness of 17.2% in contrast to 9.4% recorded previously (Asit and Pankaj 2016). Therefore, lameness occurrence might differ in the tropics due to diverse management practices influencing the predominance of associated risk factors.

Claw lesions have been reported to be majorly responsible for lameness conditions in dairy herds (Somers and O’Grady 2015; Solano et al. 2016). The disease process of claw lesions and specifically those affecting the claw horn tissues is not fully understood. Nevertheless, events such as nutritional-induced inflammation (Thoefner et al. 2004) hard floor surfaces (Bergsten et al. 2015) and physiological changes affecting the digital cushion (Newsome et al. 2017) have been shown to influence development of claw lesions. Claw lesions have also been found as subclinical affections in non-lame cows while (Tadich et al. 2010), however, reduced milk yield was reported in cows with subclinical claw lesions prior to the onset of lameness (Green et al. 2014). Hence, investigating the presence of claw lesions and clinical lameness in dairy cows is essential as the use of only locomotion scoring often underestimate the presence of claw lesions (van Nuffel et al. 2015).

In Malaysia and Selangor specifically, there is dearth information on claw health status and lameness in dairy despite recent growth in the dairy industry. Information from the large animal ward records of the institution veterinary teaching hospital reported a total of 79 lameness cases from the Ladang Angkat farms from the year 2013–2016 (Unpublished work). However, international studies have showed that farmers often underestimate lameness occurrence and present only severe cases for treatment (Horseman et al. 2014). Therefore, the objectives of this study were to investigate the prevalence of lameness, claw lesions, and associated risk factors amongst dairy herds in Selangor, Malaysia.

## Materials and methods

**Fig. 1:**
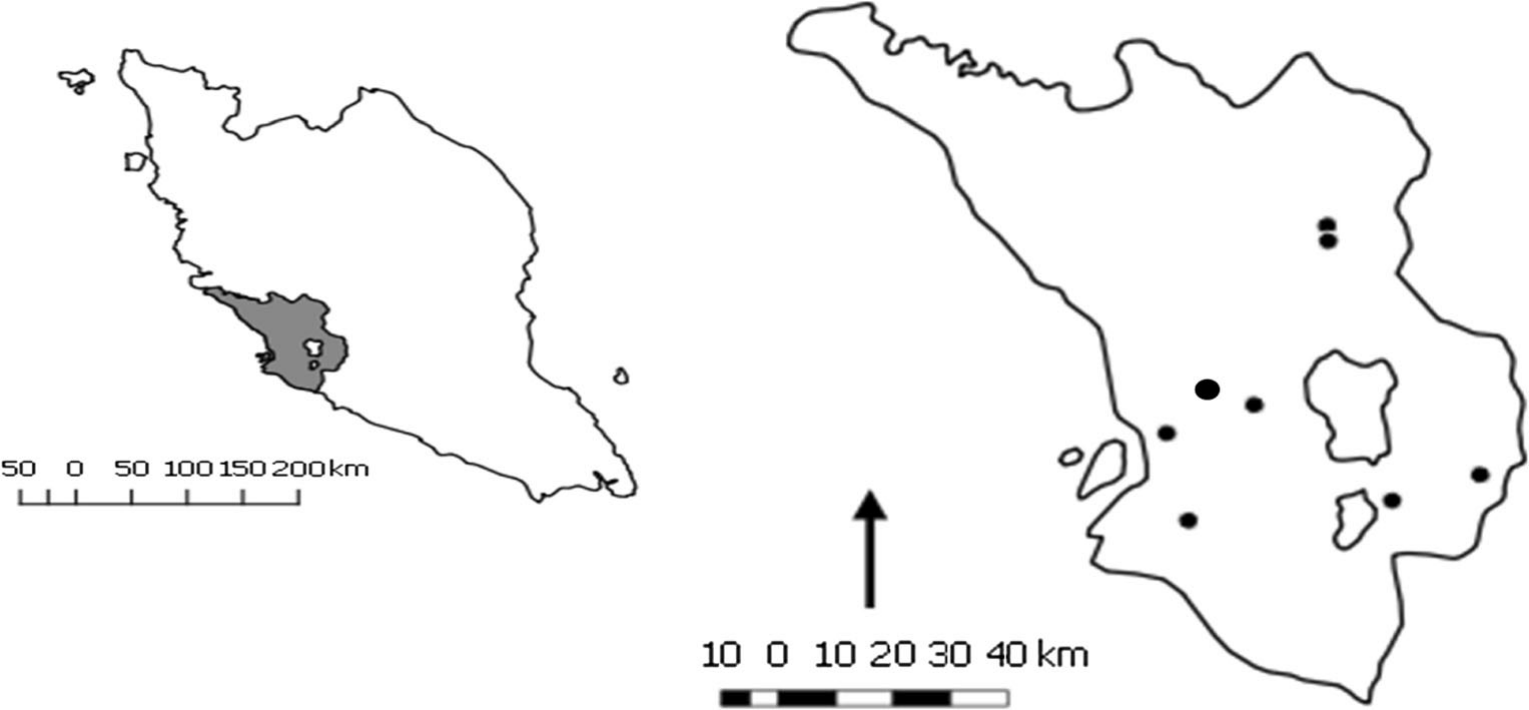
Map of Peninsular Malaysia showing the location of the study site. The enlargement of the study site indicates the location of the dairy farms (*black dots*) and the *black arrow* depicts north (Quantum GIS 2.4.0 Chugiak)

The study was carried out in Selangor on the west coast of Peninsular Malaysia, approx. 3°20′N and 101°30′E (Fig. 1). Eighteen dairy farmers registered with the Department of Veterinary Services (DVS 2014) were contacted by phone seeking their consent to participate in the study. The criteria for farm selection included intensive and free-stall management, herd size ≥ 50, and presence of 20 or more lactating cows. Eight dairy farms willing to participate and plausible for the study were selected and visited between September 2016 and February, 2017. Sample size was 251 lactating cows, a value chosen by taking into account expected lameness prevalence of 20%, confidence level of 95%, and desired precision of 5% as described by Thrusfield (2005). All the cows enrolled were the Mafriwal breed, a cross between Fresian and Sahiwal. Within each herd, 50% of all lactating cows were sampled and stratified based on parity and days in milk (DIM). Animal-based measures such as body condition score (BCS), hock condition score (HCS), and leg hygiene were recorded for each cow before assessing the locomotion score (LS).

Herd features such as herd size, number of milking cows, number of cows at early days in milk (DIM), and access to pasture were obtained from farm records and self-administered questionnaire. For management practices related to claw health, two farms had a routine claw trimming program while only one used a footbath. Also, four farms had rubber mats as stall base for individual cows.

The BCS was recorded using a 1 to 4 scale (Elanco animal health 2009) as thin, poor, moderate, and fat. Hind limbs were examined for hock condition score on 3 levels scale where, 1 = normal area with no alopecia and inflammation, 2 = hair loss but absence or slight swelling (< − 12 cm), and 3 = hair loss and substantial swelling (> 2 cm) (Gibbons et al. 2012). Leg hygiene was scored depending on the level of manure contamination of the upper and lower limbs as described by Solano et al. (2015). Lameness was assessed by using a modified 4-point locomotion score developed by DairyCo (2007) (Table 1). This involved the detailed visual examination of the posture and gait of the animal while leaving the milking palour on a flat surface**.**

**Table 1:** Locomotion scoring chart used in the study farms

Lameness score	Clinical description	Assessment criteria
1	Sound	The cow stands and walks with a level-back posture. Her gait is normal.
2	Sound	The cow stands with a level-back posture, but develops an arched-back posture while walking. Her gait is normal.
3	Lame	An arched-back posture is evident both while standing and walking. Her gait is affected and is best described as short striding with one or more limbs.
4	Lame	An arched-back posture is always evident and gait is best described as one deliberate step at a time. The cow favors one or more limbs or feet to inability to bear weight on one or more of her limbs

Source: a modified locomotion scoring system developed by DairyCo (2007)

Following restraining, the rear claw was cleaned and debris removed for clear visualization of the claw zones. Claw length was assessed using a claw check and claw angles above 45° were considered overgrown (Archer et al. 2015). Claw lesions were grouped into those affecting the hoof skin and claw horn lesions. The former consisted of digital dermatitis (DD) and heel horn erosion, interdigital hyperplasia (IH), and swelling of the coronet area (SC). Claw horn lesions included sole lesions (hemorrhage, ulcer, bruises, and double sole) (SL), white line disease (WLD), heel lesions, (HL), wall fissures (WF), overgrown claw with deformities (OC), and others (corkscrew and scissors claw) as described by Shearer and Van Amstel (2013) and the International Claw Health Atlas (2015). Different claw lesions were counted per cow whereas presence of more than one of the same lesion in a cow was recorded once.

All statistical analyses were performed using IBM SSPSS 24 (Version 24.0, IBM Corp., and Chicago, IL, USA). Data based on the assessment scoring system were screened for normality using the Shapiro-Wilk test and the distribution was non-normal. Prevalence of lameness and claw lesions was calculated as the total number of cows with LS ≥ 3 and affected with ≥ 1 or more claw lesion to the total number of observed cows in each farm. The Wilcoxon signed rank test and descriptive statistics were used to compare the prevalence estimates distribution of claw lesions, respectively. A binary logistic regression with forward procedure was applied to investigate cow level factors associated with prevalence of lameness and claw lesions. Model fit for the dataset was assessed by using the Pearson chi-square statistic and the Hosmer-Lemeshow test. Odds ratio (OR) was read from the parameter estimates at 95% confidence interval (CI) and *P* value < 0.05 was considered significant.

## Results

**Fig. 2:**
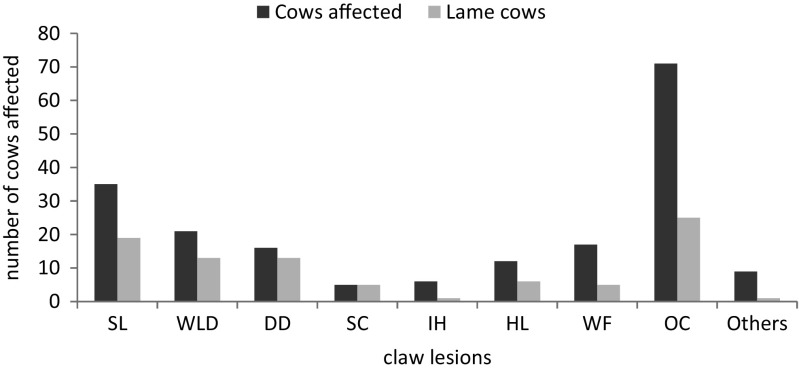
Distribution of claw lesions and the number of lame cows. Keys: SL sole lesions, WLD white line disease, DD digital dermatitis, SC swelling of the coronet area, IH interdigital hyperplasia, HL heel lesions, WF wall fissures, and OC overgrown claw with deformities

**Fig. 3:**
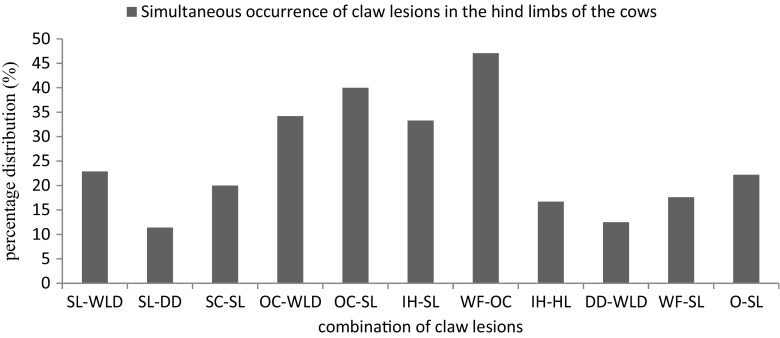
Claw lesions and their simultaneous occurrence in the dairy cows. Keys: SL sole lesions, WLD white line disease, DD digital dermatitis, SC swelling of the coronet area, IH interdigital hyperplasia, HL heel lesions, WF wall fissures, and OC overgrown claw with deformities

In all the dairy cows (251) examined, cow prevalence of lameness was 19.1% and ranged from 10.0 to 33.3% amongst the studied farms (see Table 2). Statistical test revealed a significant difference (*P* **<** 0.05) in the prevalence of lameness amongst the farms. Cow prevalence of claw lesions was 31.1% (78/251) ranging from 16.3 to 40.0% amongst the studied farms. The distribution and occurrence of claw lesions in the affected cows are presented in Table 3. A total of 192 claw lesions were recorded with the most recurrent being OC (37.0%), SL (18.2%), WLD (10.9%), WF (8.9%), and DD (8.3%). Other recorded claw lesions included SC (2.6%), IH (3.1%), HL (6.3%), and others (4.7%). The proportion of cows with single claw lesion was 28.2% whereas 71.8% had combination of either 2 or more lesions. Cows affected with WLD, SL, and DD had the highest corresponding lame cows being 61.2, 54.2, and 81.2%, respectively. However, though the occurrence of SC was low, all the affected cows were lame (Fig. 2). Overall, claw lesions were recorded in 87.5% (42/48) of the lame cows. The herd prevalence of the claw lesions showed that SL, WLD, WF, and OC were all recorded in the farms while DD was not diagnosed in 2 farms. SL was mostly found in association with OC (47.0%), IH (33.3%), WLD (23.0%), and SC (20.0%). OC was also present with WLD (34.0%) and WF (46%) while 12.0% had combined lesions of DD and WLD (Fig. 3).

**Table 2:** Prevalence of lameness and cows affected with claw lesions in the study farms based on LS and claw assessment, respectively

Farms	No. of cows	Observation	Lame cows			Claw lesions		
			No. of lame cows	%	95% CI	Cows affected	%	95% CI
F1	82	40	4	10.0	0.7–19	10	25.0	6–29
F2	34	26	5	19.2	4–34	7	26.9	7–39
F3	86	40	10	25.0	14–41	14	35.0	23–53
F4	100	49	7	14.3	5–24	11	22.4	6–27
F5	64	30	10	33.3	17–50	12	40.0	23–58
F6	38	16	2	12.5	0–29	6	37.5	14–61
F7	43	20	4	20.0	3–38	8	40.0	23–67
F8	72	30	6	20.0	6–34	10	33.3	14–46
Total	519	251	48	19.1		78	31.1	
Median ± range	68 ± 66	30 ± 33	5.5 ± 8			7 ± 8		
Wilcoxon signed rank			− 2.50		− 2.40			
*P* value			0.012*		0.011*			

*F* farms, *values* numerical notation of farms

*P* values < 0.05 are statistically significant

**Table 3:** Number of cows diagnosed with each claw lesion and corresponding number of lame cows

Claw lesions	Frequency	%	Lame cows		Non-lame cows	
			Observations	%	Observations	%
SL	35	18.2	19	54.2	16	45.8
WLD	21	10.9	13	61.9	7	38.1
DD	16	8.3	13	81.2	3	18.7
SC	5	2.6	5	100	0	0.0
IH	6	3.1	1	16.7	5	83.3
HL	12	6.3	6	50.0	6	50.0
WF	17	8.9	5	29.4	12	70.6
OC	71	37.0	25	35.2	46	64.8
Others	9	4.7	1	11.1	8	88.9
Total (%)	192	100	88 (45.8)	100	104 (54.2)	

*SL* sole lesions, *WLD* white line disease, *DD* digital dermatitis, *SC* swelling of the coronet area, *IH* interdigital hyperplasia, *HL* heel lesions, *WF* wall fissures, *OC* overgrown claw with deformity, *others* corkscrew claw and avulsion

The prevalence of lameness was not associated with parity (*P* > 0.05) whereas cows in early lactation were significantly associated (OR = 3.3; 95% CI 2–7.) with lameness occurrence compared to those in late lactation. Thin cows had the highest lameness prevalence (27.5%) but was not significantly different while fat cows (BCS > 3) were a protective factor (OR = 0.3; 95% CI 0.1–1). However, lameness was associated with injured hocks (OR = 4.8; 95% CI 5–17) as well as cows which had dirty and very dirty leg hygiene score with approximately 3 and 10 times increased odds of being lame (Table 4). Despite the higher prevalence of lameness in cows with overgrown claw (31.9%), the association tends not to be significant (*P* > 0.05). For cows with claw lesions, prevalence was associated with overgrown claw (OR = 2.7 ; 95% CI 1.4-5.3) and dirty leg hygiene score (OR = 4.7; 95% CI 4–11) (Table 5).

**Table 4:** Relationship between cow level factors and occurrence of lameness in the dairy cows using binary logistic regression

Factors	Observations (*n* = 251)	Lame cows (*n* = 48)	%	OR	95% CI	*P* value
Parity						
Primiparous	59	18	30.5	Ref		
Second parity	103	13	12.6	0.9	0.3–3	0.81
Parity 3 and above	99	27	27.2	1.6	0.6–4	0.38
DIM						
1–120 days in milk	130	33	25.3	3.3	2–7	0.003
Above 120 DIM	121	15	12.4	Ref		
BCS						
Thin	58	16	27.5	Ref		
Moderate	166	26	15.6	0.9	0.2–3	0.08
Fat	27	6	22.2	0.3	0.1–1	0.04
HCS						
Normal	170	24	14.1	Ref		
Hair loss	65	14	21.5	2.1	0.9–5	0.08
Ulcer/Swelling	16	10	62.5	4.8	5–17	0.01
HS						
Clean	133	12	9.0	Ref		
Dirty	80	18	22.5	2.6	1.3–6.2	0.04
Very dirty	38	18	47.3	9.9	4–28	0.001
Claw overgrowth						
Absent	179	25	13.9	Ref		
Present	72	23	31.9	2.0	0.9–5	0.071

*Ref* reference category

*P values < 0.05 are considered statistically significant

**Table 5:** Relationship between cow level factors and occurrence of claw lesions in the dairy cows using binary logistic regression

Factors	Observations (*n* = 251)	Cows with claw lesions	%	OR	95% CI	*P* value
Parity						
Primiparous	59	12	20.3	Ref		
2nd parity	103	27	26.2	1.7	0.7–4	0.27
Parity 3 and above	99	39	39.4	1.6	0.7–4	0.31
DIM						
1–120 days in milk	130	33	25.4	0.6	0.3–1	0.11
Above 120 DIM	121	45	37.2	Ref		
BCS						
Thin	58	24	41.4	Ref		
Moderate	166	44	26.5	0.4	0.1–1	0.12
Fat	27	10	37.0	0.2	0.1–1	0.003
HCS						
Normal	170	48	28.2	Ref		
Hair loss	65	20	30.8	1.6	0.8–3	0.23
Ulcer/Swelling	16	10	62.5	1.9	0.6–6	0.30
HS						
Clean	133	18	13.5	Ref		
Dirty	80	33	41.3	4.9	4–11	0.001
Very dirty	38	27	71.1	15.4	6–40	0.001
Claw overgrowth						
Absent	179	41	22.9	Ref		
Present	72	37	51.4	2.7	1.4–5.3	0.005

Ref = Reference category

*P values < 0.05 are considered statistically significant

## Discussion

The cow prevalence of lameness of 19.1% with herd prevalence ranging from 10 to 33% reported in this study is similar to that presented in Bangladesh and China (Rahman et al. 2014; Chapinal et al. 2014) and different from that reported in Switzerland and Ireland (Becker et al. 2014; Somers and O’Grady 2015). The disparity could be due to variation in herd size, management practices, and diagnostic criteria (Tadich et al. 2010). The present study recorded cow prevalence of claw lesions of 31.1% being responsible for 87% of the recorded lameness cases. Furthermore, OC, SL, and WLD were the recurrent claw lesions and similar to the findings from other studies (Zahid et al. 2014; Somers and O’Grady 2015). However, claw lesions other than OC were reported in other studies due to routine claw trimming practices which were lacking in most of the farms enrolled in this study. Also, the high prevalence of SL and WLD might be attributed to the hard concrete floor which exposes the solar and white line area to greater forces leading to traumatic claw lesions (Shearer 2017).

A higher proportion of cows affected with SC, DD, and WLD were lame compared to those with SL and OC which could be attributed to the distribution and simultaneous occurrence of respective claw lesions. Also, factors such as severity, stage, and duration are vital in the association between claw lesions and lameness (Palmer and O’Connell 2015; Shearer 2017). Accordingly, majority of the affected cows had more than one claw lesion in this study and consistent with other studies (Olechnowicz et al. 2011; Solano et al. 2015). The likelihood for more than one claw lesion per cow could be due to complication of primary lesions and chronic tendency of lesions such as SL, WLD, and DD (Bergsten et al. 2015; Gomez et al. 2015).

Lameness prevalence in this study was not significantly associated with BCS and parity which contradicts finding from other authors (Green et al. 2014; Solano et al. 2015). Differences might be due to system of BCS, sample size, breeds, and corresponding milk yielding capacity. However, there were significant associations between lameness prevalence and cows at early lactation, injured hocks, and dirty leg hygiene as reported by other similar studies (Bergsten et al. 2015; Relun et al. 2013; Nash et al. 2016). The structural changes in the claw capsule from hormonal effect at peri-calving period and higher milk yield were suggested to influence the increased odds of clinical lameness at early days in milk (Newsome et al. 2017). Equally, hock injuries were reported in lame cows having spent more time lying down on hard and abrasive surfaces (Nash et al. 2016). Accordingly, 5 of the studied farms had bare concrete floors which could have increased the severity of hock lesions. Likewise, exposure of the cow foot to manure slurry and poor leg cleanliness was found to enhance claw conformational changes leading to lameness (Relun et al. 2013). The association between claw overgrowth and lameness occurrence as shown herein has been demonstrated in previous studies indicating the importance of claw trimming (Solano et al. 2015). Consequently, only 2 of the studied farms practiced claw trimming. Overall, the prevalence of claw lesions was associated with dirty leg hygiene and overgrown claw. Poor leg hygiene augments the development of either infectious or claw horn lesions by exposing the feet to manure and moisture with detrimental effect on claw health (Relun et al. 2013). Other factors such as early lactation, parity, BCS, and hock condition were not associated with prevalence of claw lesions in contrary to other studies (Ranjbar et al. 2016: Solano et al. 2016). The disparity might be credited to difference in breeds and distribution of claw lesions. Nevertheless, the result herein as related to BCS and parity is consistent with results of Ristevski et al. (2016) and Olechnowicz et al. (2011), respectively.

The recorded prevalence estimates indicates that lameness and claw lesions are important health issues in the studied farms. Farmers need to improve the management of cows in early lactation, injured hock condition, leg cleanliness, and overgrown claw. Lameness occurrence and claw lesions could be reduced in dairy herds in the region based on the generated knowledge and information to the farmers and veterinarians. Further studies are required to investigate herd-level risk factors associated with lameness and specific claw lesions in dairy herds in the region.
